# The *E3 Ubiquitin* Ligase RNF7 Negatively Regulates *CARD14/CARMA2sh* Signaling

**DOI:** 10.3390/ijms18122581

**Published:** 2017-12-01

**Authors:** Gianluca Telesio, Ivan Scudiero, Maddalena Pizzulo, Pellegrino Mazzone, Tiziana Zotti, Serena Voccola, Immacolata Polvere, Pasquale Vito, Romania Stilo

**Affiliations:** 1 Biogem Consortium, Via Camporeale, 83031 Ariano Irpino (AV), Italy; luca.telesio@libero.it (G.T.); scudieroi@gmail.com (I.S.); maddalena.pizzulo@biogem.it (M.P.); pellegrinomazzone@gmail.com (P.M.); 2 Genus Biotech, Università degli Studi del Sannio, Strada Statale Appia, 82010 Apollosa (BN), Italy; titz.zotti@gmail.com; 3 Dipartimento di Scienze e Tecnologie, Università degli Studi del Sannio, Via Port’Arsa 11, 82100 Benevento, Italy; serena.voccola@virgilio.it (S.V.); stardust88@live.it (I.P.); romstilo@unisannio.it (R.S.)

**Keywords:** CARD14, CARMA2*sh*, RNF7, NF-κB, psoriasis, MALT1, ubiquitin, B-cell lymphoma/leukemia 10 (BCL10)

## Abstract

The three CARD-containing MAGUK (CARMA) proteins function as scaffolding molecules that regulate activation of the pro-inflammatory transcription factor NF-κB. Recently, mutations in CARMA2 have been linked to psoriasis susceptibility due to their acquired altered capacity to activate NF-κB. By means of two-hybrid screening with yeast, we identified RING finger protein 7 (RNF7) as an interactor of CARMA2. We present evidence that RNF7 functions as a negative regulator of the NF-κB-activating capacity of CARMA2. Mechanistically, RNF7 influences CARMA2 signaling by regulating the ubiquitination state of MALT1 and the NF-κB-regulatory molecule NEMO. Interestingly, CARMA2*short* (CARMA2*sh*) mutants associated with psoriasis susceptibility escape the negative control exerted by RNF7. In conclusion, our findings identify a new mechanism through which the ability of CARMA2 to activate NF-κB is regulated, which could have significant implications for our understanding of why mutations of this protein trigger human psoriasis.

## 1. Introduction

The CARD-containing MAGUK (CARMA) family of proteins consists of the three evolutionarily conserved proteins CARD11/CARMA1 (CARMA1), CARD14/CARMA2 (CARMA2) and CARD10/CARMA3 (CARMA3) [1,2]. CARMA proteins share a similar structure, consisting of an N-terminal CARD domain followed by a coiled-coil (CC) domain, a linker region, and a C-terminal membrane-associated guanylate kinase domain (MAGUK), which in turns comprises PSD-95/Dlg1)/ZO-1 (PDZ), SRC Homology 3 Domain (SH3), and guanylate kinases (GUK) subdomains [1,2]. Functionally, CARMA proteins behave as scaffolding molecules that can activate the pro-inflammatory transcription factor NF-κB [1,2]. More specifically, the CARD domains of CARMA proteins interact with the CARD domain of B-cell lymphoma/leukemia 10 (BCL10), which binds the protease Mucosa-associated lymphoid tissue lymphoma translocation protein 1 (MALT1). The resulting CARMA–BCL10–MALT1 (CBM) complex then mediates downstream signaling, including the recruitment and activation of the IKK complex and activation of the NK-κB pathway [1,2,3]. While CARMA1 and CARMA3 play an essential role in NF-κB signaling induced by antigen receptors and certain G-protein-coupled receptors (GPCRs), respectively [1,2], in human keratinocytes CARMA2 plays an indispensable role in the signal transduction pathway that links pathogen-associated molecular pattern recognition to NF-κB activation [4]. Recent works have shown that mutations in CARMA2 segregate with several human inflammatory skin disorders, including familial and non-familial cases of psoriasis vulgaris, psoriatic arthritis, generalized pustular psoriasis, palmoplantar pustular psoriasis, and pityriasis rubra pilaris [5,6,7,8,9,10,11,12,13,14,15,16,17,18]. Many of the CARMA2 mutations associated with human skin disorders result in an increased activity of NF-κB transcription factor [2,4,5,19].

Similar to CARMA1 and CARMA3, activation of NF-κB promoted by CARMA2 is also mediated by the CBM complex [20,21,22]. Thus, to identify novel regulators of CARMA2 signaling, we carried out two-hybrid screening using CARMA2*short* (CARMA2*sh*) [22] as a bait. We identified RING finger-containing protein 7 (RNF7) as a novel CARMA2 interactor. We show evidence that RNF7 negatively regulates CARMA2-mediated NF-κB signaling, whereas a psoriasis-linked CARMA2 mutant escapes this negative regulation.

## 2. Results

In order to identify novel interactors of CARMA protein, a set of two-hybrid screenings were carried out in the yeast reporter strain AH109. When CARMA2*sh*, the CARMA2 isoform predominantly expressed in human keratinocytes [5,23] was used as a bait, several clones were isolated in multiple independent copies encoding for the RING finger protein 7 (RNF7), a 113-amino acid protein also referred to as the sensitive to apoptosis gene (SAG) [24,25], regulator of cullins 2 (ROC2) or RING-box 2 (Rbx2). RNF7 was originally identified as a redox-inducible anti-oxidant protein [24] and is also a component of E3 ubiquitin ligases with E3 ubiquitin ligase activity [26]. Subsequent experiments carried on with one of these library plasmids confirmed the interaction of RNF7 with CARMA2*sh* in the reporter yeast strain (Table 1).

**Figure 1:**
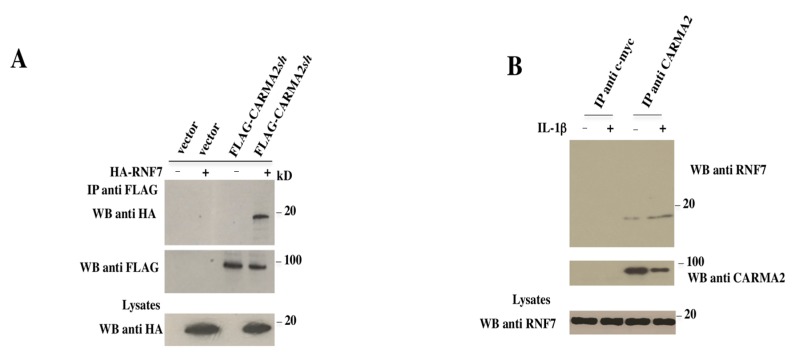
RNF7 binds to CARMA2*sh*. (**A**) HEK293T cells were cotransfected with a plasmid encoding for HA-tagged RNF7 together with a FLAG-tagged expression vector empty or encoding for CARMA2*sh*. Twenty-four hours later, lysates were immunoprecipitated with anti-FLAG antibodies and analyzed for coprecipitating HA-RNF7 by Western blot assay. (**B**) HaCaT cells were left untreated or stimulated with IL-1β for 30 min. Cell lysates where then prepared, immunoprecipitated with anti-RNF7 or anti-myc control antibodies, and analyzed for coprecipitating CARMA*sh* by Western blot assay. IP: Immunoprecipitation; WB: Western Blot.

To test for a direct association between CARMA2*sh* and RNF7 in mammalian cells, HEK293T cells were cotransfected with plasmids expressing FLAG-tagged CARMA2*sh* together with a vector encoding for HA-tagged RNF7 (Figure 1A). Cell lysates were immunoprecipitated with anti-FLAG-coated beads, and the presence of coprecipitating RNF7 was monitored by immunoblot experiments with anti-HA antibody. The result shown in Figure 1A indicates that CARMA2*sh* coprecipitates with RNF7 in transfected cells. Next, we tested whether CARMA2*sh* and RNF7 endogenously interact in the HaCaT keratinocytic human cell line, which expresses both proteins. Indeed, as shown in Figure 1B, when protein lysates extracted from HaCaT cells where immunoprecipitated with an anti-CARMA2 antibody but not the control antibody anti c-myc, coprecipitating RNF7 was detected by an immunoblot assay. Interaction of RNF7 with CARMA2*sh* slightly increased following stimulation of HaCaT cells with Interleukin 1 beta (IL-1β).

**Figure 2:**
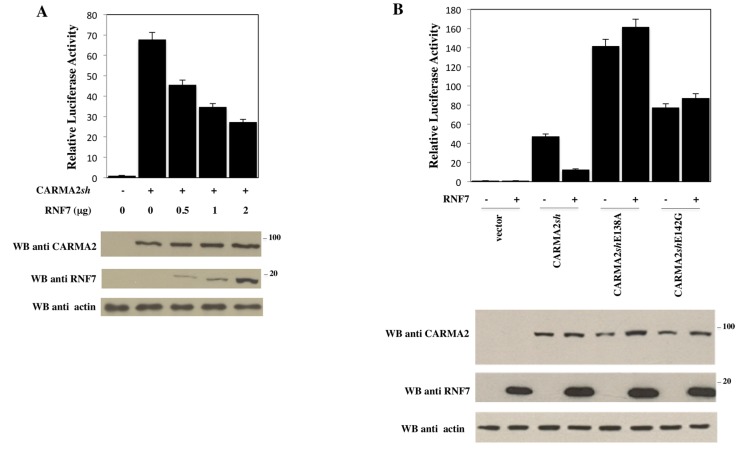

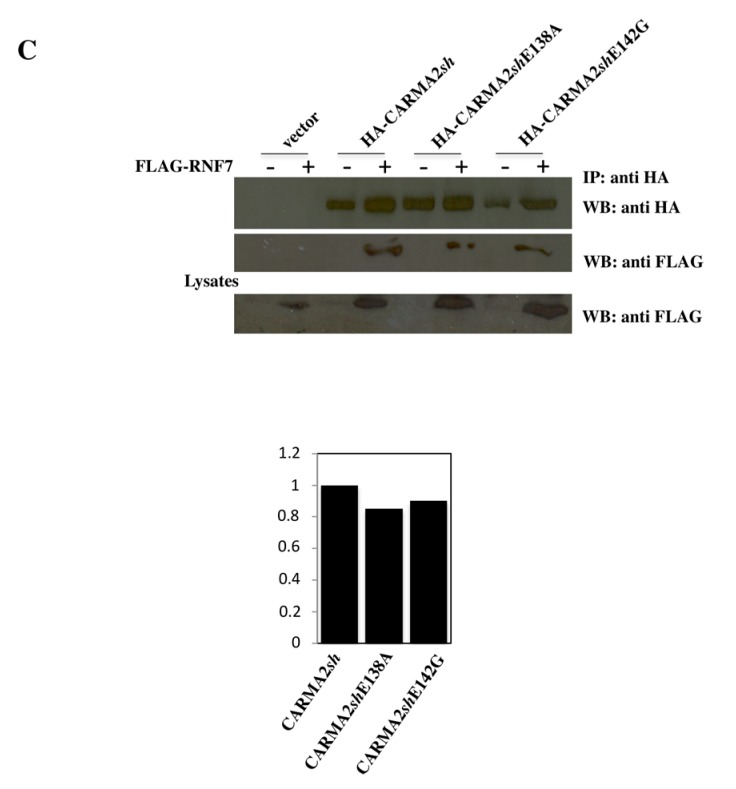
RNF7 represses the NF-κB-inducing activity of CARMA2*sh*. (**A**) HEK293T cells were transiently cotransfected with expression vectors encoding for CARMA2*sh* (1 μg) and RNF7 in the indicated amount, together with NF-κB-luciferase and β-galactosidase reporter vectors. Twenty-four hours after transfection, cell lysates were prepared and luciferase activity was measured. Data shown represent relative luciferase activity normalized against β-galactosidase activity and are representative of at least ten independent experiments done in triplicate. Lower panel: A fraction of the cell lysate was analyzed by immunoblot assay to monitor protein expression. (**B**) HEK293T cells were transiently cotransfected as in (**A**) with expression vectors encoding for wild type (wt) or psoriasis-linked mutants of CARMA2*sh* (1 μg), with or without a vector encoding for RNF7. The total amount of transfected DNA was kept constant by adding empty vector. Twenty-four hours after transfection, cell lysates were prepared and luciferase activity was measured. Data shown represent relative luciferase activity normalized against β-galactosidase activity and are representative of at least 10 independent experiments done in triplicate. Lower panel: A fraction of the cell lysate was analyzed by immunoblot assay to monitor protein expression. (**C**) Upper panel: HEK293T cells were cotransfected with a plasmid encoding for HA-tagged RNF7 together with a FLAG-tagged expression vector empty or encoding for wt CARMA2*sh* or psoriasis-associated mutant forms of CARMA2*sh*. Twenty-four hours later, lysates were immunoprecipitated with anti-FLAG antibodies and analyzed for coprecipitating HA-RNF7 by Western blot assay. Lower panel: Densitometric analysis of RNF7 associated to wt and mutant forms of CARMA2*sh*. IP: Immunoprecipitation; WB: Western Blot.

Since CARMA2*sh* is implicated in the NF-κB signaling pathways, we then investigated for an involvement of RNF7 in this pathway using a luciferase-based reporter assay. Indeed, as shown in Figure 2A, RNF7 significantly represses, in a dose-dependent manner, NF-κB activation elicited by CARMA2*sh* expression. Interestingly, the NF-κB activation produced by the psoriasis-linked mutants CARMA2*sh*E138A and CARMA2*sh*E142G were not affected by RNF7 expression (Figure 2B), despite both mutants still being associated with RNF7 (Figure 2C).

**Figure 3:**
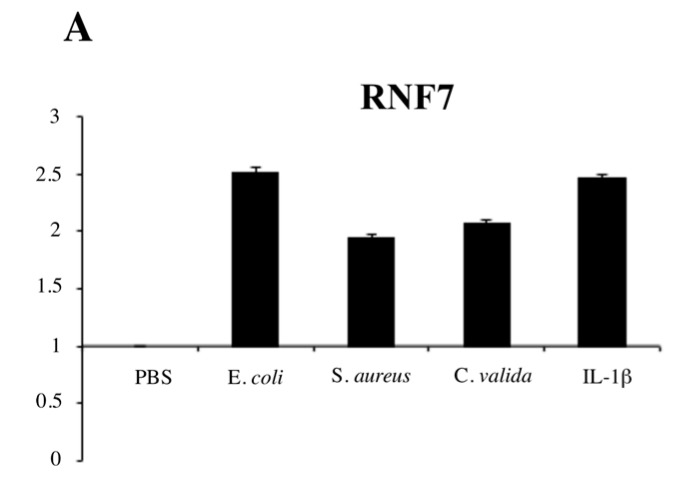

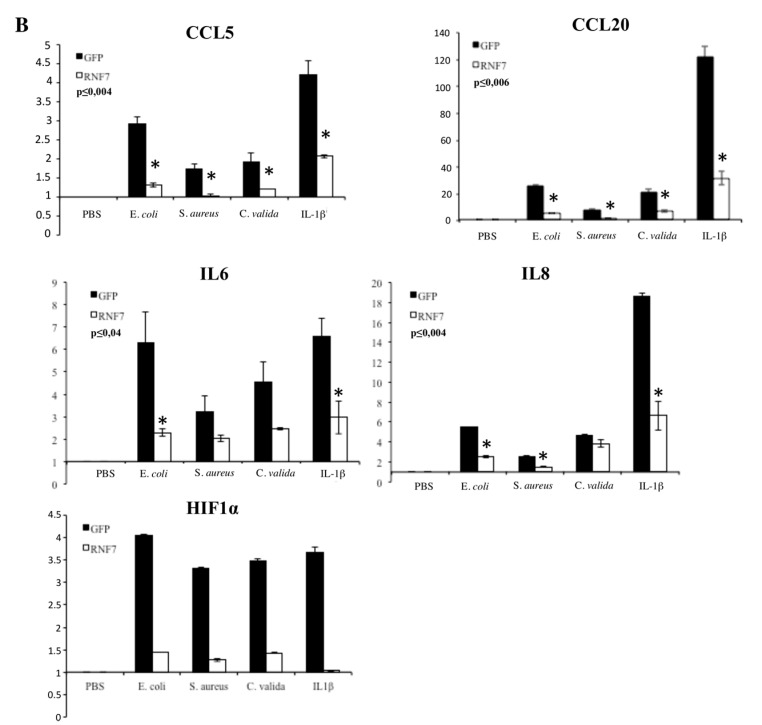
RNF7 expression represses NF-κB signaling upon pathogen-associated molecular pattern (PAMP) recognition. (**A**) HaCaT cells were left in phosphate-buffered saline (PBS) or exposed to the indicated heat-killed microorganisms or IL-1β (10 ng/mL) for 6 h, and the expression level of RNF7 was monitored by real-time PCR. Graph show the fold changes respect to the cells left in PBS. Data shown is representative of three independent experiments done in triplicate. (**B**) HaCaT cells were infected with a lentiviral vector expressing RNF7 or control GFP. Forty-eight hours later, cells were left in PBS or exposed to the indicated heat-killed microorganisms for 6 h, and the expression levels of selected NF-κB target genes were monitored by real-time PCR. Data shown represents the fold changes respect to the GFP-transfected cells left in PBS. Data were analyzed by Student’s *t*-test, and a *p*-value ≤ 0.05, indicated with an * was considered significant. Data shown is representative of at least three independent experiments done in triplicate.

We have previously shown that in human keratinocytes CARMA2*sh* plays a key role in the signal transduction pathway that connects pathogen-associated molecular pattern (PAMP) recognition to NF-κB activation [4]. In fact, exposure to PAMPs activates NF-κB following agonistic binding to pattern recognition receptors, which includes members of the toll-like receptor family expressed on human keratinocytes [27]. Interestingly, we noted that RNF7 expression is induced in HaCaT cells exposed to heat-killed bacterial (*E. coli* and *S. aureus*) or fungi (*C. valida*) cells or the inflammatory cytokine IL-1β (Figure 3A).

Thus, we tested whether expression of RNF7 influences NF-κB activation following PAMP exposure. For this, HaCaT cells were infected with a retroviral vector encoding for RNF7 or control GFP, and the induction of several NF-κB target genes following exposure to heat-killed microorganisms or IL-1β was monitored by real-time PCR. Indeed, as shown in Figure 3B, expression of RNF7 significantly reduces the induction of all the NF-κB target genes assessed.

**Figure 4:**
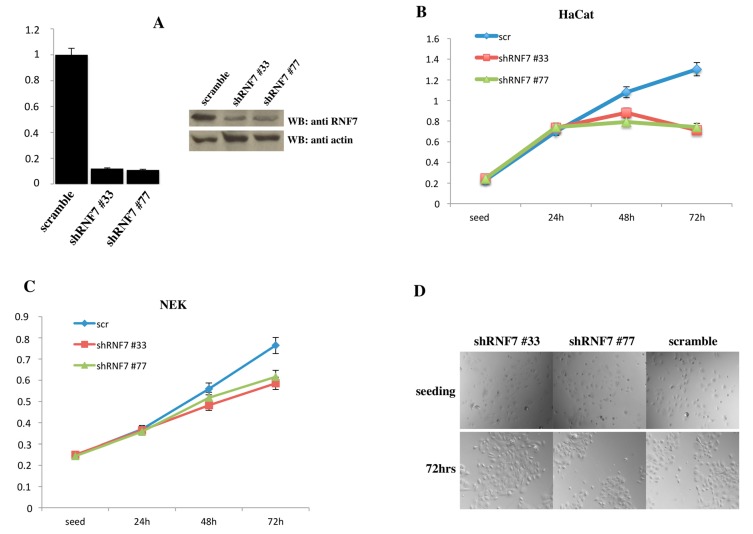
RNF7 depletion results in cell death (**A**) Left panel: HaCaT cells were infected with retroviral vectors encoding for two different short hairpin RNAs (shRNAs) targeting RNF7 or a control shRNA (scramble). After selection, RNF7 mRNA levels normalized to glyceraldehyde 3-phosphate dehydrogenase (GAPDH) were quantified by real-time PCR. Right panel: In the same cells RNF7 expression was monitored by immunoblot assay. (**B**) HaCaT cells and (**C**) human normal epidermal keratinocytes were infected with a lentiviral vector expressing a scramble sequence or shRNAs targeting RNF7 and cell viability was monitored at the indicated time points by 3-(4,5-dimethylthiazol-2-yl)-2,5-diphenyltetrazolium bromide (MTT) assay. (**D**) 20× phase contrast micrographs of the human normal epidermal keratinocytes used in the experiment shown in (**C**). NEK: normal epidermal keratinocytes.

We next tried to abrogate RNF7 expression in HaCaT cells and primary human normal epidermal keratinocytes (NEK) using short hairpin RNAs targeting human RNF7. By abolishing RNF7 expression, in fact, we would expect a greater NF-κB response following PAMP stimulation. However, depletion of RNF7 expression in both HaCaT and NHEK resulted in a marked reduction of cellular viability (Figure 4), indicating that RNF7 expression is required for survival in cultured cells.

**Figure 5:**
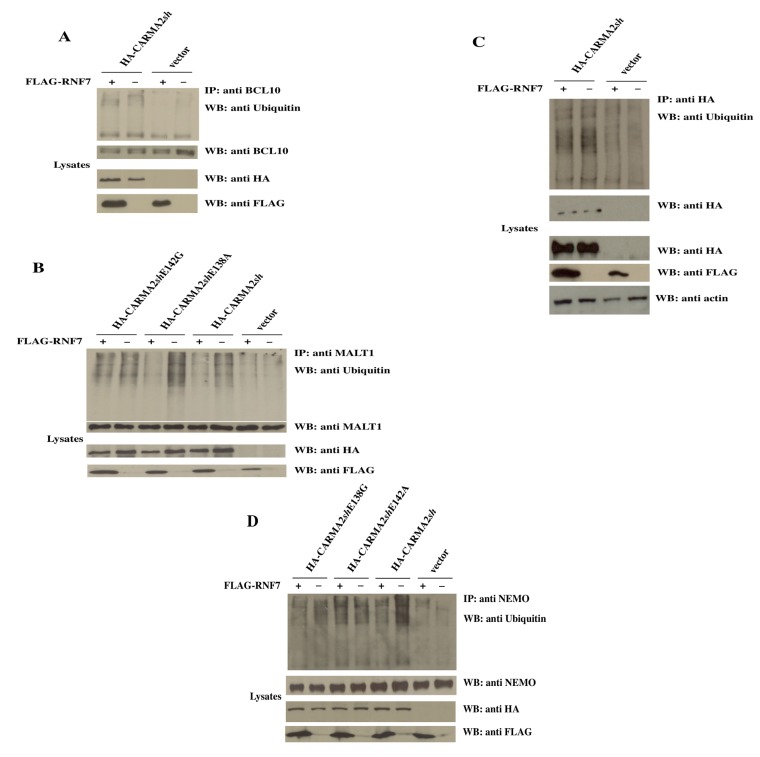
RNF7 controls the ubiquitination state of NF-kappa-B essential modulator (NEMO) and Mucosa-associated lymphoid tissue lymphoma translocation protein 1 (MALT1). HEK293T cells were cotransfected with the indicated expression vectors. Twenty-four hours later, lysates were prepared and immunoprecipitated with (**A**) anti-BCL10, (**B**) anti-HA, (**C**) anti-MALT1, and (**D**) anti-NEMO antibodies. Immunocomplexes were separated by SDS-PAGE and blotted on membranes subsequently probed with anti-ubiquitin antibody.

Since RNF7 is a ubiquitin ligase, and ubiquitination events play an important role in the CBM-mediated activation of the NF-κB pathway [1], we investigated whether RNF7 could modify such ubiquitination reactions. In fact, as shown in Figure 5A, transfection of CARMA2*sh* in HEK293T cells results in ubiquitination of BCL10, and the simultaneous expression of RNF7 does not interfere with that ubiquitination event. Similarly, RNF7 does not alter the ubiquitination state of CARMA2*sh* (Figure 5B), which has been reported as one of the mechanisms through which the function of CARMA protein is regulated [28]. However, RNF7 significantly reduces the ubiquitination of MALT1 and NF-kappa-B essential modulator (NEMO) induced by CARMA2*sh* expression (Figure 5B,C). Interestingly, RNF7 abrogates ubiquitination of MALT1 and NEMO induced by the psoriasis-associated mutant CARMA2*sh*E138A, but not that induced by the psoriasis-associated mutant CARMA2*sh*E142G.

## 3. Discussion

Long known as the least characterized of the three CARMA proteins, the recently discovered involvement of CARMA2 in inflammatory diseases of human skin has spurred intense research on this protein. Thus, recent data has shown that similarly to CARMA1 and CARMA3, the NF-κB signaling triggered by CARMA2 requires the BCL10 and MALT1 proteins organized in the CBM complex [4,20,21,22]. Also, similarly to CARMA1 and CARMA3, NF-κB signaling induced by CARMA2 is abrogated by the de-ubiquitinase A20 [20], implying that ubiquitination events are also involved in the regulation of CARMA2 function. In this work we have identified RNF7 as a CARMA2*sh*-associated molecule capable of negatively regulating its ability to activate NF-κB transcription factor. This data is of particular significance since most of CARMA2 mutants associated with psoriasis are characterized by promoting a deregulated, increased activation of NF-κB [2,19]. It is therefore interesting to note that RNF7 is able to repress the NF-κB-inducing activity of wild type (wt) CARMA2*sh*, but not that of the two psoriasis-associated mutants CARMA2*sh*E138A and CARMA2*sh*E142G.

**Figure 6:**
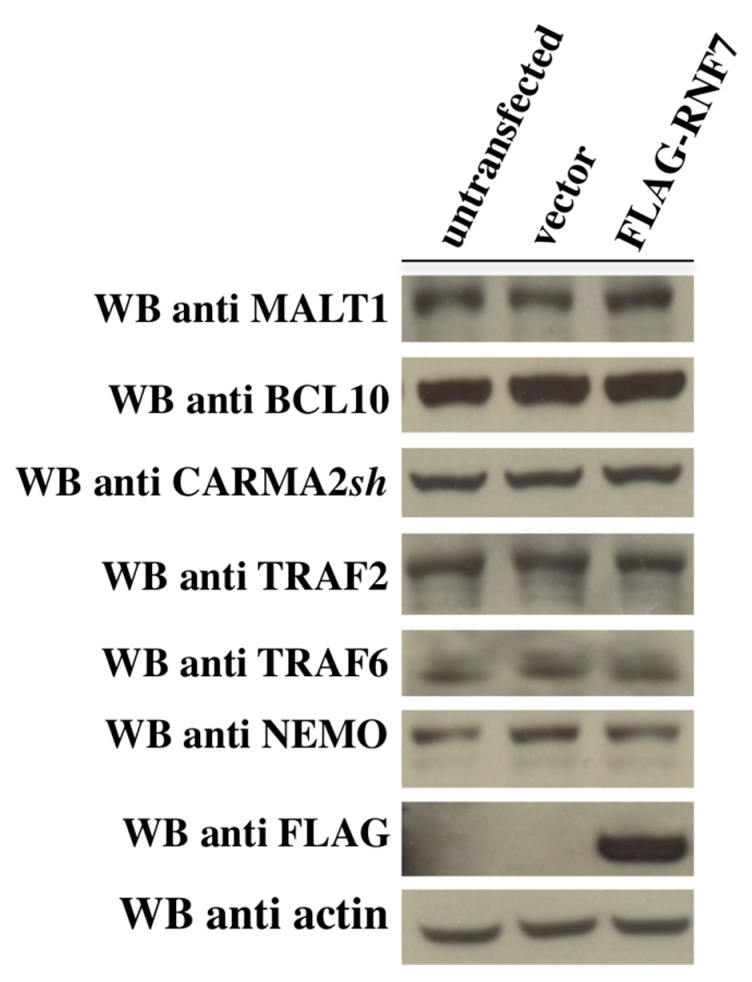
The CARMA–BCL10–MALT1 (CBM) complex expression level was not affected by RNF7. HEK293T cells were left untransfected or transfected as indicated. Twenty-four hours later, lysates were prepared and the expression level of the indicated proteins was monitored by immunoblot assay. CARMA: CARD-containing MAGUK.

Our data is consistent with a recent paper by Pedersen et al., showing that RNF181, a protein belonging to the same family as RNF7, negatively regulates induction of NF-κB mediated by CARMA1 in lymphoid cells [29]. However, the mechanisms through which RNF181 and RNF7 modulate CARMA proteins signaling seem to be different. In fact, RNF181 controls CBM-mediated NF-κB signaling by regulating the expression level of BCL10 protein. However, in our experiments, the expression levels of BCL10, CARMA2*sh*, MALT1, and key NF-κB regulators such as TRAF2, TRAF6, and NEMO are not affected by RNF7 expression (Figure 6 and data not shown).

In our experiments, RNF7 expression promotes deubiquitination of MALT1 and NEMO, which is a well-known post-translational modification that affects activation of NF-κB mediated by the CBM complex [30,31,32,33]. Thus, it appears that there are multiple mechanisms employed by RNF proteins to regulate CARMA proteins function. RNF7 possess a E3 ubiquitin ligase activity [26], and seems to be unlikely that just a RING finger protein, like RNF7, can mediate a deubiquitination reaction. This evidence clearly indicates that the deubiquitination of MALT1 and NEMO promoted by RNF7 occurs through an indirect mechanism, raising the fascinating possibility that RNF7 may target for degradation an upstream molecule(s) responsible for ubiquitination of MALT1 and NEMO. That intriguing scenario, though, remains to be demonstrated.

Finally, a particular comment should be made on the behavior of the CARMA2*sh* mutants associated with psoriasis analyzed in this study. In fact, while with regard to NF-κB activation both mutants CARMA2*sh*E138A and CARMA2*sh*E142G are insensitive to the negative control exercised by RNF7 (Figure 2B), they behave differently with regard to MALT1 ubiquitination. In fact, RNF7 abrogates ubiquitination of MALT1 induced by CARMA2*sh*E138A expression, but not MALT1 ubiquitination induced by CARMA2*sh*E142G expression (Figure 4B), indicating that the various CARMA2 mutants associated with psoriasis so far identified can cause the disease through different molecular mechanisms. Much work is still needed to decipher these mechanisms.

## 4. Materials and Methods

### 4.1. The Two-Hybrid Screening

The two-hybrid screening carried out using CARMA2*sh* as a bait as described in [34]. Briefly, yeast strain AH109 GAL4^−/−^ was first transformed with pGBKT7 plasmids carrying a CARMA2*sh* cDNA bait fused with DBD of GAL4 using the lithium acetate/PEG 3000 procedure. Transformant colonies were selected on synthetic dropout plates lacking tryptophan. Expression of bait fusion proteins was assessed by immunoblot analysis. For library screening, yeast AH109 expressing GAL4DBD–CARMA2*sh* was transformed with a human fetal brain cDNA library cloned in pACT2 vector (Clontech) in fusion with GAL4TAD. Then, 2 × 10^6^ clones were screened for interaction with GAL4DBD–CARMA2*sh* using selective growth on minimal medium lacking nutrients for which biosynthesis is mediated by genes under the control of GAL4 transcriptional activity.

### 4.2. Cell Culture and Tranfection

HEK293T and HaCaT cells were obtained from the ATCC and cultured in Dulbecco’s modified Eagle’s medium supplemented with 10% FCS. Normal human epidermal keratinocytes (NHEKs) were purchased from Lonza and cultured according to the provided instructions. HEK293T cells were transfected by calcium phosphate precipitation; NHEKs were transfected using DreamFect Gold Transfection Reagent (OZ Biosciences Inc., Marseille, France) according to the manufacturer’s instruction. HaCaT were transfected with Lipofectamine^®^ 3000. Retroviral infections were carried out as previously described [35,36].

### 4.3. Immunoblot Analysis and Coprecipitation

Cell lysates were made in lysis buffer (150 mM NaCl, 20 mM HEPES, pH 7.4, 1% Triton X-100, 10% glycerol) and a mixture of proteases inhibitors (Protease Inhibitor Cocktail, Roche) according to the manufacturer’s instructions. Proteins were separated by SDS–PAGE, transferred onto nitrocellulose membrane, and incubated with primary antibodies followed by horseradish peroxidase-conjugated secondary antibodies (Amersham Biosciences Corp., Piscataway, NJ, USA). Blots were developed using the enhanced chemiluminescen (ECL) system (Amersham Biosciences Corp.). For co-immunoprecipitation experiments, cells were lysed in lysis buffer and immunocomplexes were bound to protein A/G (Amersham Biosciences) for 2 h at 4 °C. Immunocomplexes were extensively washed, resolved by SDS–PAGE, and analyzed by immunoblot assay. Antisera and monoclonal antibodies were the following: anti-FLAG, anti-β-Actin, (Sigma-Aldrich, St. Louis, MI, USA); anti-HA, anti-myc, anti-MALT1, anti-CARMA2, anti-NEMO, anti-ubiquitin, (Santa Cruz Biotechnology, Dallas, TX, USA); and anti-RNF7 (Abcam, Cambridge, UK). The anti-BCL10 antibody was described in [37].

### 4.4. Luciferase and β-Galactosidase Assays

To assess for NF-κB activation, cells were co-transfected in 6-well plates with 0.2 μg of pNF-κB-luc (Clontech Laboratories, Mountain View, CA, USA) and 0.1 μg of pRSV-βGal (Addgene, Cambridge, MA, USA) plus each expression plasmid. When necessary, the total amount of transfected plasmidic DNA (2 μg) was kept constant by adding empty vector. pNF-κB-luc encodes the firefly luciferase reporter gene under the control of a minimal (m) CMV promoter and tandem repeats of the NF-κB transcriptional response element. The plasmid RSV-βGal, expressing β-galactosidase, was added to the transfection mixture in order to normalize for the efficiency of transfection. After transfection and treatments, luciferase activity was determined with Luciferase Assay System (Promega, Madison, WI, USA). For measurement of β-galactosidase activity, 20 μL of cell lysates diluted 100-fold with 0.1 M potassium phosphate buffer were mixed with 200 μL of Galactone (Tropix, Bedford, MA, USA) that were diluted 100-fold with 0.1 M potassium phosphate and 1 mM magnesium chloride, pH 7.8, for 1 h at room temperature. Then, β-galactosidase activity was measured after addition of 300 μL of Emerald (Tropix). Luciferase activity was normalized on β-galactosidase activity and expressed in arbitrary units.

### 4.5. Real-Time RT-PCR

Total RNA was isolated from cells or tissues using TRIzol reagent (Invitrogen, Carlsbad, CA, USA). The reverse transcriptase reaction was performed using 1 μg of total RNA in a 20 μL reaction and 1 μL of the resulting cDNA was used in the subsequent amplification step along with 300 nM of each primer. The geometric mean values of β-actin and succinyl-CoA synthetase β-subunit fragment were used as normalization factors. The relative transcription level was calculated by using the ΔΔ*C*_t_ method. Real-time PCR reactions were performed in triplicate by using the SYBR Green PCR Master Mix (Qiagen, Venlo, The Netherlands) in a 7900HT system (Applied Biosystems, Foster City, CA, USA).

### 4.6. MTT Assay

At the end of each testing time, the culture supernatants were removed, MTT solution (0.5 mg/mL) was added to each well, and the plates were incubated for 1 h at 37 °C. The MTT solution was removed, and isopropyl alcohol was added to dissolve formazan crystals. The absorbance at 570 nm was read on a microplate spectrophotometer (Applied Biosystem). shRNA targeting RNF7 were obtained from Sigma and have the following sequence:

TRCN0000298777 (#77):
  CCGGCCTGTGGGTGAAACAGAACAACTCGAGTTGTTCTGTTTCACCCACAGGTTTTTG
TRCN0000295933 (#33):
  CCGGGTAATCCAGTGCCCTACAAAGCTCGAGCTTTGTAGGGCACTGGATTACTTTTTG.

## Figures and Tables

**Table 1:** Interaction of CARMA2*short* (CARMA2*sh*) with RING finger protein 7 (RNF7) in the yeast two-hybrid assay.

Protein Fused to GAL4 Domain		Yeast Growth on Selective Media
DNA-Binding	Activating	
-	RNF7	−
Vector	RNF7	−
Fas-associated protein with death domain (FADD)	RNF7	−
CARMA2*sh*	RNF7	+
CARMA2*sh*	-	−

Yeast AH109 was transformed with CARMA2*sh* fused to the GAL4-DNA binding domain together with the indicated cDNAs fused to the GAL4-activating domain. The cDNA encoding for FADD served as a putative negative control. Interactions were examined by yeast growth on selective media; assays were done for 10 independent transformants. Yeast colonies were scored as positive (+) when growth developed within 24–36 h; a negative (−) was scored when growth failed to develop within 1 week.
